# Patients attended by palliative care teams: are they always comparable populations?

**DOI:** 10.1186/2193-1801-2-177

**Published:** 2013-04-22

**Authors:** Maria Nabal, Miquel Barcons, Roberto Moreno, Xavier Busquets, Javier J Trujillano, Antonio Requena

**Affiliations:** 1 Family and Community Medicine, Palliative Care Support Team, Arnau de Vilanova University Hospital, ICS Institut de Reserca Biomédica de Lleida (IRBL), Lleida, Spain; 2 Family and Community Medicine, Home Care Support Team (PADES) Granollers, ICS, Granollers, Spain; 3 Family and Community Medicine Home Care Support Team (ESAD) Zaragoza SALUD, Aragon Health Sciences Institute, Zaragoza, Spain; 4 Department of Basic Medical Sciences, Faculty of Medicine, University of Lleida, Institut de Reserca Biomédica de Lleida (IRBL), Lleida, Spain; 5 Family and Community Medicine, Medical Emergency Unit, Huesca SALUD, Aragon Health Aragon Sciences Institute, Kragujevac, Spain; 6 Ufiss Cuidados Paliativos, Hospital Universitario Arnau de Vilanova, Av. Rovira Roure 80, Lleida, 25198 Spain

## Abstract

Patients attended by palliative care teams: are they always comparable populations? To answer this question we have compared the basic epidemiological characteristics of patients attended by home palliative care teams (HPCT) in two autonomous regions of Spain.

We carried out a coordinated analytical, observational and prospective study in two Spanish autonomous regions: Aragon and Catalonia. Data were kept during each home care visit according to patients' needs. Inclusion criteria were: advanced cancer, over 18 years old and first contact with a HPCT. The recruitment period was 6 months. Variables included were: Survival time (days), age, sex, primary disease and extension, place of residence. Functional and cognitive state, and co-morbidity. 10 signs/symptoms: asthenia, anorexia, cachexia, dysphagia, xerostomy, dyspnoea, oedemas, level of consciousness, presence of delirium, presence of pressure ulcers and some treatment data. Others variables considered were: responsible team, origin, destination when discharge, date and place of death, number of visits made and duration of monitoring. We developed a comparison between groups by Chi-squared test or the non-parametric Mann–Whitney U test and a survival analysis by Kaplan-Meier curves and the logrank test to determine differences between factors. The SPSS version 15.0 software package was used.

698 patients were included, 56.2% from Aragon and 43.8% from Catalonia. 60.3% were males, without differences between the regions. Characteristics relative to age, sex, place of residence and extension of oncological diseases were similar for both groups. We found significant differences between the two populations relative to survival time, co-morbidity, functional state, presence and intensity of a number of symptoms and the treatments, patient monitoring and the their destination after discharge.

We can conclude that palliative care teams cover different profiles of patients with regard to their co-morbidity, functional, cognitive and symptomatic states. It must be pointed that the organization of palliative care services and their experience appears to condition the profile of patients they attend. There is a need of consensus on the basic descriptors for palliative care patients in order to ensure that results will be comparable.

## Introduction

Cicely Saunders started the modern hospice movement in 1967 with the founding of St Christopher’s Hospice in London (Clark 2000). The development of palliative care in Europe and in Spain has progressed slowly since then (SECPAL 2012; Rocafort 2007).

The European Association for Palliative Care (EAPC) carried out a study on the development of palliative care in European countries. The Task Force on the Development of Palliative Care in Europe, headed by Carlos Centeno and David Clark, published its results in the EAPC Atlas Of Palliative Care In Europe (Centeno et al. 2007). For the first time it offered reliable information that enabled the state of palliative care services in the different European countries to be compared (Rocafort & Centeno 2008). This study illustrated the existence of a number of common organizational structures and great diversity in the development of different types of programmes and in the provision of services. These differences are, at least, partly related to the different ways of interpreting the underlying concepts and terms in palliative medicine. In order to make valid comparisons, it is necessary to develop a common language (von Guten 2007).

With this aim, the EAPC published its suggestions for use in Europe of a common terminology following a process of consensus with national associations. Quality standards will be defined based on this terminology of consensus. Guidelines for regulations and standards are necessary not only for health care professionals working in the field of palliative care, but also for those entrusted with taking health-related decisions, who are responsible for providing patients with suitable access to palliative care (Radbruch et al. 2009; Radbruch et al. 2010).

The differences that exist in the organizational framework are transferred to the clinical framework (Currow et al. 2009). Do palliative care teams attend the same type of patients? Can comparisons be made between patients in different regions of the same country or between those of different countries? Does the degree of development of palliative care services influence the characteristics of the patients they attend? There are currently no clear answers to these questions, and although a number of initiatives exist that are trying to reach consensus on the minimum information required to describe some symptoms, there is as yet no standardized practice (Jack et al. 2009; 2010; Knudsen et al. 2009). This fact has great transcendence in research and clinical practice. If populations are not homogeneous, the results of multicentric studies may not be completely conclusive. Will we be able to transfer the results of research carried out in other countries to our patients without local validation?

Therefore, it would seem necessary to seek consensus on the basic descriptors that characterize patient samples, simultaneously with the carrying out of studies that verify similarities and differences in groups of patients attended by different teams in different geographical or cultural areas and/or different countries.

Catalonia and Aragon are two neighbouring autonomous regions with uneven trajectories in palliative care.

Catalonia is an autonomous region with an area of 31,895 km^2^ located in the north-east of the Iberian Peninsula. It has a population of 7,364,079, which accounts for 15.9% of Spain’s total. The demographic distribution is uneven, oscillating between 1.3 and 17,725 inhabitants per km^2^. Palliative care services in Catalonia started in 1987 (Centeno et al. 2000) and the regional government of Catalonia produced a pilot plan for the development of palliative care services in 1990 in partnership with the World Health Organization (Sanz 1999; Porta Sales & Albo 1998). This institutional support led to the development of a global palliative care network (Gómez-Batiste et al. 1992): 134 specialist palliative care teams, 59 of which act as home care teams, 28 as hospital support teams and 33 as PCUs in university teaching hospitals and medium-term and long-term care facilities. There are also 6 psychosocial teams and 2 centres that act as observatories (Gómez-Batiste et al. 2007).

The autonomous region of Aragon is also located in the north-east of the Iberian Peninsula. It is the fourth largest of Spain’s autonomous regions in size, with an area of 47,720 km^2^; however, its low population density of barely 25 inhabitants per km^2^ is the second lowest in the country. The population of the region does not exceed 1,200,000, although its distribution is closely associated with the location of industry and services. Practically half of the population of Aragon lives in its capital, Zaragoza. Palliative care services began in Aragon in 1990, as the result of the concern felt by a group of primary care practitioners who created the first palliative care protocol in a health centre (Torrubia et al. 1990). The first publicly financed home care support team was set up in 1999 (Instituto Nacional de la Salud 1999). The approval of the National Palliative Care Plan in 2000 fostered the progressive development of new resources (Gobierno de Aragón 2003). Aragon currently has 11 specialist palliative care teams, 8 of which act as home care teams and 1 as a hospital support team; 1 PCU in a medium and long-term care facility; and 1 psychosocial team 2.

A study carried out by the Spanish Ministry of Health established that Catalonia was an autonomous region with an equitable distribution of resources and access times for both home and hospital palliative care. However, Aragon does not have an equitable distribution of resources or isochronal intervention, despite great efforts made to provide home care services in rural areas (Herrera et al. 2012).

This study was developed with the aim of establishing similarities and differences between patients attended by palliative care teams in two Spanish autonomous regions as part of a broader study on prognostic tools in palliative care (Nabal et al. 2010a; Nabal et al. 2010b).

## Material and methods

This study forms part of a broader study for the validation and improvement of prognostic models in palliative care. It is therefore a coordinated analytical, observational and prospective study. The recruitment period was 6 months, with monitoring until the patients’ death or 180 days.

The data collected came from all visits made by each palliative care team according to the needs of each patient.

The study was carried out in Aragon and Catalonia. The work was carried out by 8 home care support teams (ESAD) in Aragon and by 5 home care support teams (PADES) in Catalonia.

Sample size: A sample size of 693 patients was defined. The sample was calculated according to published recommendations of 10–15 events for each dichotomous variable, and 10–15 (n-1) for each categorical variable.

Inclusion criteria: patients with advanced cancer, over 18 years of age and first contact with a palliative home care team.

The analysed variables were:

- Survival time (days).
- General information: age, sex, primary disease and extension, place of residence.
- Clinical information: functional state (Karnofsky, Barthel, ECOG), cognitive state (Pfeiffer), co-morbidity (Charlson) and 10 signs/symptoms rated according to their intensity by a categorical scale between 0 a 3 where 0 was non, 1 was slight, 2 was moderate and 3 was severe. The sings and symptoms assessed were: asthenia, anorexia, cachexia, dysphagia, dry mouth, dyspnoea, oedemas, level of consciousness, presence of delirium, presence of pressure ulcers.
- Treatment-related information: corticosteroids, subcutaneous butterfly needle.
- Activity information: responsible team, origin, destination when discharge, date and place of death, number of visits made and duration of monitoring.

The categorical variables are expressed as a percentage and the quantitative variables as mean ± standard deviation. The comparison between groups was made by means of the Chi-squared test or the non-parametric Mann–Whitney U test according to the variable type.

Survival analysis was made using with Kaplan-Meier curves and the logrank test to determine differences between factors. Statistical significance was defined as p < 0.05.

The SPSS version 15.0 software package was used.

## Results

**Figure 1:**
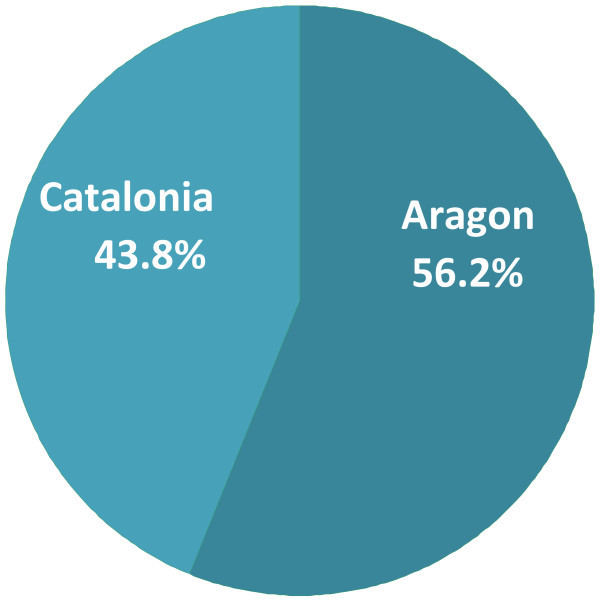
**Sample distribution by region.**

698 patients were included, of which 56.2% were from Aragon and 43.8% were from Catalonia. 60.3% were males, without differences between the regions (Figure 1).

Table 1 shows the general clinical and demographic characteristics of the patients. The sample did not show significant differences by age, gender, place of living or cancer type and stage. The clinical characteristics relative to functional and cognitive state, co-morbidity and treatment can be seen in Table 2. It can be pointed that performance status was better for those patients treated in Catalonia by any performance status scale used. No differences were found on cognitive status or co – morbidity. The use of corticoids and subcutaneous butterfly was greater in Aragon, which may be related to the worse performance status of this sample.

**Table 1:** **Clinical and demographic characteristics of patients (n = 698)**

	TOTAL	ARAGON	CATALONIA	p-value^b^
	(n = 698)	(n = 392)	(n = 306)	
Age (years) ^a^	**73.7 ± 12**	**74.9 ± 11**	**72.1 ± 13**	**0.002**
Gender, male (%)	**60.3**	**59.6**	**61.1**	**0.694**
Urban (%)	**69.8**	**66.8**	**73.7**	**0.049**
Metastasis (%)	**62.5**	**64.8**	**59.5**	**0.150**
Neoplasm (%)				**0.013**
**Head and Neck**	**3.2**	**2.6**	**3.9**	
**CNS**	**25.3**	**25.1**	**25.5**	
**Upper Digestive tract**	**17.7**	**20.1**	**14.7**	
**Low Digestive tract**	**19.9**	**17.7**	**22.5**	
**Lung**	**15.6**	**16.1**	**15.0**	
**Genitourinary**	**6.9**	**7.4**	**6.2**	
**Breast**	**1.8**	**0.5**	**3.3**	
**Haematological**	**2.3**	**3.7**	**0.7**	
**Others**	**3.8**	**3.7**	**3.9**	
**Unknown**	**3.5**	**2.9**	**4.2**	

^a^: mean ± standard deviation; ^b^: median (interquartile range). ^b^: Comparison between groups; p determined by the χ^2^ test for Comparison of proportions or Mann–Whitney test for continuous variables.

**Table 2:** **Functional and cognitive state, co-morbidity and treatment (n = 698)**

	TOTAL	ARAGON	CATALONIA	p-value^b^
	(n = 698)	(n = 392)	(n = 306)	
KARNOFSKY^a^	**51.8 ± 14**	**48.8 ± 15**	**55.7 ± 13**	**< 0.001**
ECOG^a^	**2.5 ± 1**	**2.7 ± 1**	**2.2 ± 1**	**< 0.001**
BARTHEL^a^	**56.2 ± 33**	**49.5 ± 34**	**64.9 ± 30**	**< 0.001**
PFEIFFER^a^	**2.6 ± 4**	**2.9 ± 4**	**2.1 ± 4**	**0.094**
CHARLSON^a^	**5.8 ± 2**	**5.9 ± 2**	**5.6 ± 2**	**0.020**
Corticosteroids (%)	**37.5**	**29.8**	**47.4**	**< 0.001**
SC Butterfly needle (%)	**8.3**	**9.9**	**6.2**	**0.076**

SC: Subcutaneous. ^a^: mean ± standard deviation. ^b^: Comparison between groups; p determined by the χ^2^ test for Comparison of proportions or Mann–Whitney test for continuous variables.

Table 3 shows the similarities and differences related to signs and symptoms. As can be seen globally, the proportion of patients suffering from anorexia, asthenia, dry mouth, dyspnoea or dysphagia did not differ between both places of care; although the highest levels of anorexia, asthenia and dry mouth were more frequent in Aragon. When focusing on signs, Oedemas and level of conscience, were worse in Aragon but higher levels of cachexia and pressure sores were more frequent in Catalonia.

**Table 3:** **Comparative analysis of symptoms (n = 698)**

	TOTAL	ARAGON	CATALONIA	p-value^a^
	(n = 698)	(n = 392)	(n = 306)	
Anorexia (%)				**0.010**
**0**	**29.1**	**25.8**	**33.3**	
**1**	**33.0**	**35.2**	**30.1**	
**2**	**27.8**	**26.3**	**29.7**	
**3**	**10.2**	**12.8**	**6.9**	
Asthenia (%)				**0.114**
**0**	**16.5**	**16.4**	**16.7**	
**1**	**31.3**	**29.5**	**33.7**	
**2**	**32.8**	**31.5**	**34.3**	
**3**	**19.4**	**22.6**	**15.4**	
Dry mouth (%)				**0.246**
**0**	**41.4**	**41.2**	**41.6**	
**1**	**34.6**	**32.2**	**37.7**	
**2**	**17.5**	**19.7**	**14.8**	
**3**	**6.5**	**6.9**	**5.9**	
Oedemas (%)				**0.366**
**0**	**66.8**	**64.3**	**69.9**	
**1**	**21.1**	**23.0**	**18.6**	
**2**	**9.6**	**9.7**	**9.5**	
**3**	**2.6**	**3.1**	**2.0**	
Consciousness (%)				**0.886**
**0**	**88.7**	**89.0**	**88.2**	
**1**	**6.7**	**6.1**	**7.5**	
**2**	**3.2**	**3.3**	**2.9**	
**3**	**1.4**	**1.5**	**1.3**	
Dyspnoea (%)				**0.106**
**0**	**59.3**	**63.3**	**54.2**	
**1**	**18.3**	**16.3**	**20.9**	
**2**	**12.6**	**10.5**	**15.4**	
**3**	**7.7**	**7.9**	**7.5**	
**4**	**2.0**	**2.0**	**2.0**	
Dysphagia (%)				**0.788**
**0**	**76.3**	**76.7**	**75.7**	
**1**	**13.5**	**12.5**	**14.8**	
**2**	**5.5**	**6.1**	**4.6**	
**3**	**2.4**	**2.6**	**2.3**	
**4**	**2.3**	**2.0**	**2.6**	
Cachexia (%)				**0.268**
**0**	**52.9**	**56.1**	**48.9**	
**1**	**28.4**	**26.0**	**31.5**	
**2**	**13.3**	**13.0**	**13.8**	
**3**	**5.3**	**4.8**	**5.9**	
Pressure sores (%)				**0.117**
**0**	**90.7**	**88.9**	**93.0**	
**1**	**6.9**	**8.5**	**4.7**	
**2**	**1.3**	**1.8**	**0.7**	
**3**	**0.6**	**0.5**	**0.7**	
Delirium (%)	**4.6**	**5.6**	**3.3**	**0.142**

^a^Comparison between groups; p determined by the χ^2^ test.

Finally, Table 4 shows the results for activity: responsible team, origin, destination after hospital discharge, date and place of death, number of visits made and duration of monitoring. The number of patients treated by palliative home care teams coming form general practitioners or oncologist were greater in Aragon. On the other hand, the number of patients discharged form palliative care units to home palliative care teams was higher in Catalonia, which may be related to the greater number of PCU in this region. In the same way, the time of follow up was longer in Catalonia. It must be pointed out that patients treated in Aragon got off PC program more than twice comparing with Catalonia.

**Table 4:** **Palliative care team activity (n = 698)**

	TOTAL	ARAGON	CATALONIA	p-value^b^
	(n = 698)	(n = 392)	(n = 306)	
Point of origin				**0.002**
**Primary care**	**55.0**	**56.6**	**52.9**	
**Oncology**	**22.6**	**25.0**	**19.6**	
**Other departments**	**13.3**	**11.2**	**16.0**	
**PCU**	**4.6**	**2.0**	**7.8**	
No. of visits^a^	**7 ± 4**	**5 ± 3**	**8 ± 5**	**< 0.001**
Duration on programme^a^ (days)	**65 ± 64**	**51 ± 55**	**77 ± 68**	**< 0.001**
Situation (%)				**< 0.001**
**Death**	**74.4**	**72.4**	**76.8**	
**Off programme**	**16.9**	**22.4**	**9.8**	
**Alive**	**8.7**	**5.1**	**6.2**	

^a^: mean ± standard deviation; ^b^: Comparison between groups; p determined by the χ^2^ test for Comparison of proportions or Mann–Whitney test for continuous variables.

Regarding the place of death, significant differences were seen (p < 0.001). In Aragon the proportion of patients dead at home was greater (78 vs. 41%). On the other hand, comparing the number of patients died at PCU, this was greater in Catalonia (44 vs. 4%). No significant differences were fond on the percentage of patients who died at acute hospitals between Aragon and Catalonia (18 vs. 15%).

**Figure 2:**
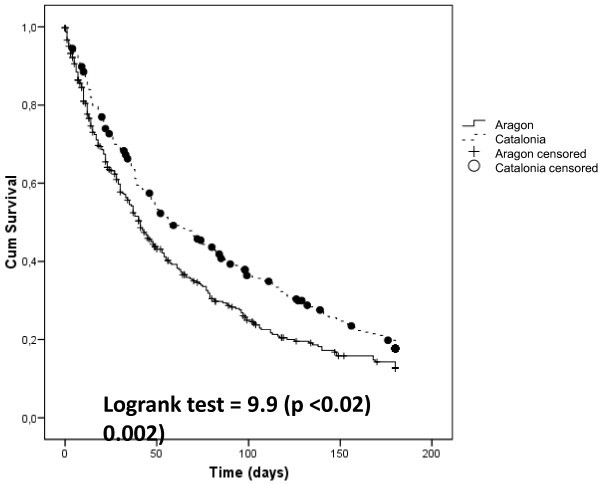
**Survival curves.**

The survival time for both groups is expressed as Kaplan-Meier curves in Figure 2 and was greater in Catalonia.

## Discussion

This work illustrates the differences that exist in the characteristics of patients attended by home palliative care teams in two different neighbouring regions in Spain. This work is part of broader research that has the aim of establishing the prognostic values of different symptoms and the impact of repeated measurements on prognostic models (Christakis & Iwashyna 2000; Passik et al. 2004).

Despite the limitations that can arise from a methodology that is not specially designed to establish similarities and differences between these two population groups, the findings obtained invite the reflection. The study involves a broad sample of patients included consecutively. Recruitment was the result of home palliative care services provided by the 13 participating teams. The breadth of the inclusion criteria may justify part of the epidemiological variability, but it does not explain the differences that exist between the two regions. Nevertheless, following along the lines of the work by Currow et al. (2009), our research compiled parameters of co-morbidity, primary disease and functional state, together with basic information on age and sex. However, information related to psychosocial factors were not considered, although the fact that patients remained in their homes is already an indirect factor for requiring basic support.

The patients involved were treated by palliative care teams and had different survival times, a difference that has been maintained over time in favour of Catalonia. This may be explained by the greater development of palliative care services in Catalonia, which began in 1990. The intervention by the Catalan teams seems to be made earlier during the course of the disease, ensuring more prolonged monitoring. This coincides with Currow et al. (2009), who assert that there might be factors related to professional training and staffing that influence patient profiles and which should be described in order for results to be correctly interpreted (Christakis & Iwashyna 2000). Passik et al. (2004) and Rowett et al. (2009) draw attention to the fact that health policy choices influence access to and the development of palliative care services.The fact that health policies are the responsibility of each autonomous region may contribute to enhance the differences. As Christakis points out, in many cases, access to palliative care services is very late and the differences in survival times are seen to be influenced by the primary disease but also by the type of care to which they have access (Christakis & Escarce 1996).

In contrast, the Aragonese population presented a higher mean age, worse functional and cognitive states, and higher rates of co-morbidity, although the differences in this last parameter were not significant. These data may explain the different survival times for the two groups, given that many authors have correlated functional state and co-morbidity with a poor prognosis (Nabal et al. 2002). The existence of differences in survival times of cancer patients between countries has been extensively discussed and seems to bear relation to early diagnosis and the treatments offered (Coleman et al. 2011).

In relation to symptoms, we identified that the prevalence and intensity of symptoms such as asthenia, cachexia and dyspnoea is higher in Catalonia in comparison with Aragon, which presents higher frequencies and intensity in delirium, dry mouth, anorexia and oedemas. Taking into account that mortality is higher in Aragon, the data gathered leads to the suspicion that patients suffering from dyspnoea or intense caquexia leave their homes or do not return to them. The profile of patients attended in Aragon seems to be less complex on the whole from a clinical perspective, but with greater difficulty for access to and attention by palliative care services due to their greater geographical dispersion and the lower number of specialist resources.

Intervention for both groups is made mostly at the request of a general practitioner, particularly in the case of the Catalan population where more than half of the intervention requests came from primary care. It seems that the integration of home palliative care support teams is more consolidated in Catalonia, and it is more common to find general practitioners and home palliative care teams working together. Among the other points of origin, it is of note that patients in the Catalan group come from PCUs, whilst this group is much reduced in Aragon. This could be explained by the fact that there is a support network in Catalonia that covers almost the entire region at all levels of health care, while Aragon has a model based on home care with few long-stay PCUs that is mostly based in large urban areas (Nabal et al. 2010b).

The patients in our sample from Aragon are less commonly located in an urban area, which means that their access to long-stay units is more difficult and less frequent.

There has been growing interest in recent years in describing the organization of palliative care services in different countries (Centeno et al. 2007; Burt et al. 2010; Lynch et al. 2009; Ferris et al. 2009), although populations treated by palliative care teams have not been so accurately compared. Most of the scientific works published in this regard dedicate a section to the description of the population covered by them, but there is no consensus on the minimum essential data, making it difficult to compare the results of such research, or else we run the risk of extrapolating results that are applicable to us to any profile of patient treated by palliative care teams (Currow et al. 2009).

In fact, these findings can be extended to the description of the main symptoms treated in palliative care. The absence of consensus, on the basic descriptors for pain, makes comparison between studies difficult. The systematic reviews concerning cachexia and pain demonstrate that the absence of shared definitions and the different idiomatic connotations attached to descriptors make difficult to generalize the results of research (Knudsen et al. 2009; Fearon et al. 2011; Blue et al. 2011).

There is currently an EAPC research network initiative in progress, in collaboration with the PRISMA project and the European Palliative Care Research Centre (PRC) in Trondheim, Norway, to reach consensus on the minimum set of data capable to describe the population receiving palliative care. This is currently being developed using the Delphi method.

Our work corroborates the need for international consensus in which descriptors for co-morbidity, cognitive and functional state, and the presence or absence of the most prevalent symptoms are contemplated in addition to personal details and particulars of the disease. These data should possibly be complemented with information on the emotional and social/family sphere of patients and the intervention setting. We will only be able to define homogeneous groups and compare the results of our research with a more detailed profile of our patients.
